# Inflammatory Mediator Profiles Differ in Sepsis Patients With and Without Bacteremia

**DOI:** 10.3389/fimmu.2018.00691

**Published:** 2018-04-06

**Authors:** Knut Anders Mosevoll, Steinar Skrede, Dagfinn Lunde Markussen, Hans Rune Fanebust, Hans Kristian Flaatten, Jörg Aßmus, Håkon Reikvam, Øystein Bruserud

**Affiliations:** ^1^Department of Clinical Science, University of Bergen, Bergen, Norway; ^2^Department of Medicine, Haukeland University Hospital, Bergen, Norway; ^3^Department of Cardiology, Haukeland University Hospital, Bergen, Norway; ^4^Department of Clinical Medicine, University of Bergen, Bergen, Norway; ^5^Centre for Clinical Research, Haukeland University Hospital, Bergen, Norway

**Keywords:** adhesion molecules, bacteremia, cytokine, hierarchical clustering, matrix metalloproteases, sepsis

## Abstract

Systemic levels of cytokines are altered during infection and sepsis. This prospective observational study aimed to investigate whether plasma levels of multiple inflammatory mediators differed between sepsis patients with and those without bacteremia during the initial phase of hospitalization. A total of 80 sepsis patients with proven bacterial infection and no immunosuppression were included in the study. Plasma samples were collected within 24 h of hospitalization, and Luminex^®^ analysis was performed on 35 mediators: 16 cytokines, six growth factors, four adhesion molecules, and nine matrix metalloproteases (MMPs)/tissue inhibitors of metalloproteinases (TIMPs). Forty-two patients (52.5%) and 38 (47.5%) patients showed positive and negative blood cultures, respectively. There were significant differences in plasma levels of six soluble mediators between the two “bacteremia” and “non-bacteremia” groups, using Mann–Whitney *U* test (*p* < 0.0014): tumor necrosis factor alpha (TNFα), CCL4, E-selectin, vascular cell adhesion molecule-1 (VCAM-1), intracellular adhesion molecule-1 (ICAM-1), and TIMP-1. Ten soluble mediators also significantly differed in plasma levels between the two groups, with *p*-values ranging between 0.05 and 0.0014: interleukin (IL)-1ra, IL-10, CCL2, CCL5, CXCL8, CXCL11, hepatocyte growth factor, MMP-8, TIMP-2, and TIMP-4. VCAM-1 showed the most robust results using univariate and multivariate logistic regression. Using unsupervised hierarchical clustering, we found that TNFα, CCL4, E-selectin, VCAM-1, ICAM-1, and TIMP-1 could be used to discriminate between patients with and those without bacteremia. Patients with bacteremia were mainly clustered in two separate groups (two upper clusters, 41/42, 98%), with higher levels of the mediators. One (2%) patient with bacteremia was clustered in the lower cluster, which compromised most of the patients without bacteremia (23/38, 61%) (χ^2^ test, *p* < 0.0001). Our study showed that analysis of the plasma inflammatory mediator profile could represent a potential strategy for early identification of patients with bacteremia.

## Introduction

Sepsis was defined as a systemic response to infection often leading to organ dysfunction and organ failures (1–3). A new definition published defines sepsis “as a life-threatening organ dysfunction caused by a dysregulated host response to infection” (4). Sepsis is an important cause for hospitalization and mortality. The inflammatory response in sepsis has been studied extensively over the last decades (3–6), including the role of cytokine profiles and signaling molecules in predicting clinical outcomes in sepsis (7–9). Bacteremia has been associated with disease severity in patients with sepsis in an intensive care unit (ICU) setting; in univariate analysis, bacteremia has also been associated with mortality, although not after correction for organ failure and early appropriate antimicrobial therapy (10–12).

Recognition of pathogens by pathogen-recognizing receptors initiates a complex inflammatory response through interactions between the pathogen, circulating immunocompetent cells, endothelial cells, and extravascular cells (13). Cytokines are important regulators of inflammation where they induce the expression of adhesion molecules on endothelial cells for leukocyte binding to the endothelium in the initial event of the inflammatory process. This is followed by leukocyte migration into the surrounding tissue, in which chemokines play an essential role for the further migration into the tissue and matrix metalloproteases (MMPs) act as important local modulators of inflammation (13–16). MMPs exert pro-inflammatory effects through proteolytic cleavage and subsequent activation of cytokines, as well as inducing the release of biologically active soluble adhesion molecules that inhibit leukocyte binding to membrane-bound adhesion molecules (17–19). Thus, these various biological soluble mediators form a dynamic and interactive network that regulates the process of inflammation.

The majority of previous studies focused on the role of single cytokines and adhesion molecules, and are reviewed elsewhere (5, 15, 20, 21). The emergence of multiplex testing has enabled the study of the wider inflammatory response, and this novel technique has been suggested as a potential diagnostic tool for sepsis by better characterizing specific subsets of patients with sepsis (4). Several studies of systemic cytokine profiles in patients with sepsis admitted to ICUs demonstrated that certain patterns correlate with sepsis severity, organ failure, and mortality (8, 9, 22–24). Similar findings have been found in a study from patients from the emergency department where broader biomarker profiles that show predictive value for severe sepsis, septic shock, and death (25). Two other studies revealed only weaker or no associations between sepsis and cytokine profiles, respectively (7, 26). Previous studies also proposed that systemic responses/profiles of soluble mediators could be used for diagnostic or prognostic evaluation of patients with inflammatory disorders (e.g., malignant diseases, stem cell transplantation, and venous thrombosis) (27–29). Sepsis is an inflammatory response as part of the clinical/systemic response to infection and give clinical signs such as fever, elevated heart rate/respiratory rate, lover blood pressure, redness, and swelling. The previous definition of sepsis has been based on the recognition of a clinical/systemic response together with an infection (1, 3, 4). Sepsis patients with such inflammatory responses are very heterogeneous highly dependent on clinical characteristics (host, microbe, infection site, etc.), and new studies to further decipher this heterogeneity is needed.

The previous studies of cytokine profiles did not include soluble adhesion molecules, MMPs or protease inhibitors in their final analyzes, neither patient cohorts from the emergency department that focuses on differences in type of infection (Gram-positive versus Gram-negative) and the severity of intravascular bacterial load (bacteremia versus non-bacteremia) (7–9, 22–26). In the present study, we characterized the biological heterogeneity in terms of inflammatory mediator profiles in a consecutive group of emergency department patients admitted with sepsis using Luminex^®^ and unsupervised hierarchical clustering analyzes.

## Materials and Methods

### Study Population

**Figure 1:**
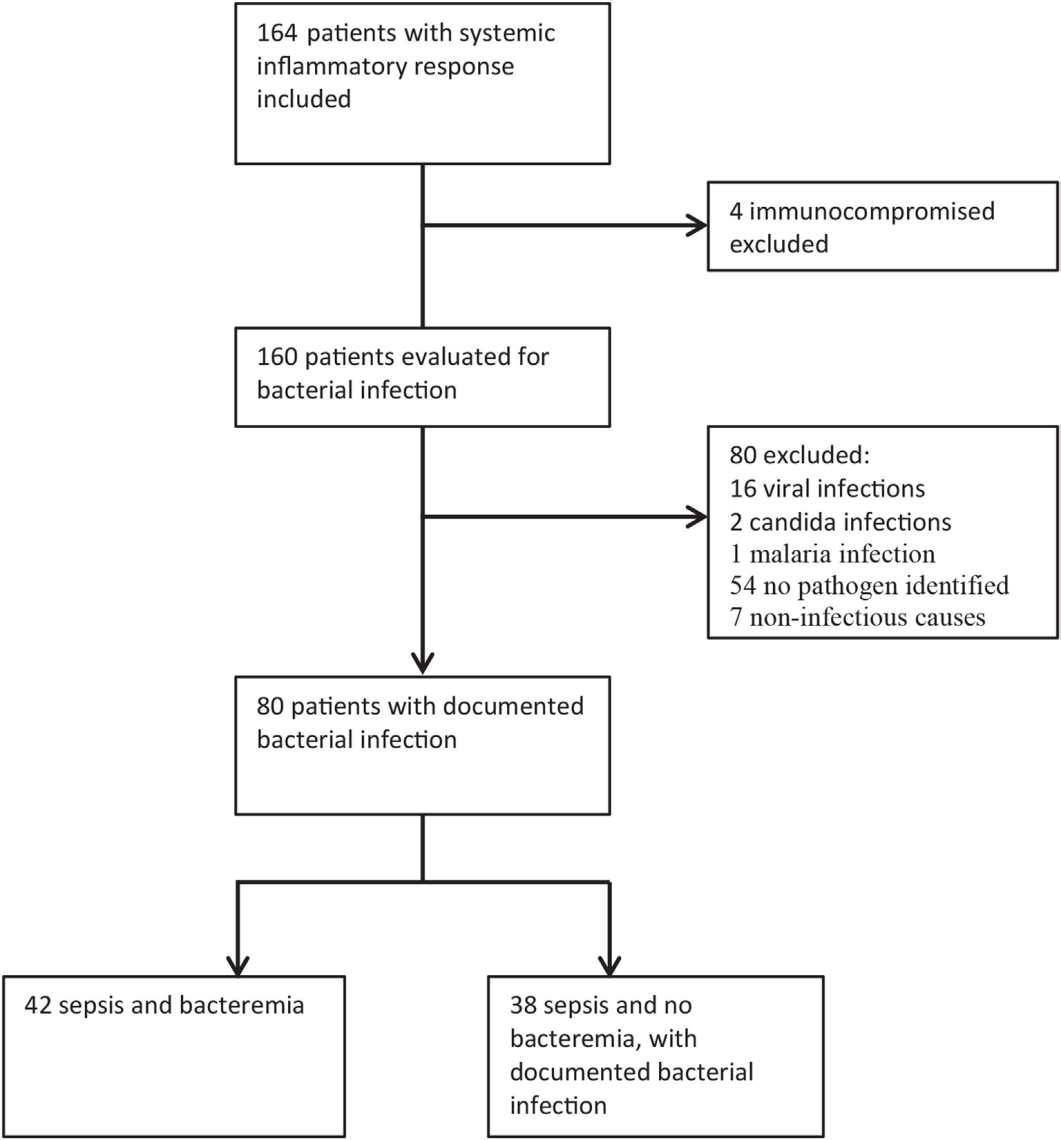
Patient enrollment and exclusion; the total number of included patients with systemic inflammatory response and the identification of immunocompetent patients with bacterial infections are shown in the figure. This study is based on the old sepsis definition, while the patients fulfilling the new sepsis definition is discriminated in Figure 4 [total sequential (sepsis-related) organ failure assessment ≥2].

This was a prospective study conducted at Haukeland University Hospital, which is a tertiary hospital in western Norway that also functions as a local hospital for approximately 300,000 inhabitants. Patients aged 16 years or more who were admitted with sepsis to the emergency department at Haukeland University Hospital between December 2012 and 2014 were included. Of a total of 164 consecutive patients admitted with clinical sepsis, 80 were immunocompetent patients with a later documented bacterial infection (Figure 1). We excluded patients with viral and parasitic infections, those without proven infections, immunocompromised patients, i.e., those with known congenital or acquired (e.g., human immunodeficiency virus infection) immunodeficiency, as well as those receiving immunosuppressive/cytotoxic treatment. All included patients provided written consent for study participation. The study was approved by the regional Ethics Committee (REK Vest Norway, number 103-2013), and conducted in accordance with the ethical standards laid down in the 1964 Declaration of Helsinki and its later amendments.

### Case Definition

In this study, sepsis was defined as the presence of infection as well as the presence of at least two of the following criteria as part of the systemic inflammatory response syndrome: (i) temperature >38°C or <36°C; (ii) heart rate >90 beats per minute; (iii) respiratory rate >20 breaths/minute or partial pressure of carbon dioxide in arterial blood (PaCO_2_) <32 mm Hg; and (iv) white blood cell count >12 × 10^9^/L, <4 × 10^9^/L, or >10% band forms (1–3). We also included the new sepsis definition by identifying those patients with an infection and an increase in total sequential (sepsis-related) organ failure assessment (SOFA) score of ≥2 (4). Serious individual organ failures were defined as sepsis-induced failures with an increase in SOFA score of ≥2; stable pre-existing organ failures were not included (3, 4).

### Plasma Samples and Blood Cultures

Plasma samples were collected within 24 h (within 3 h in the majority of cases) of hospitalization, and a minimum of two sets of blood cultures were taken before treatment with antibiotics was commenced. Blood was collected in Vacutainer 9NC tubes (Becton-Dickinson, San Jose, CA, USA) containing citric acid as anticoagulant, and then transferred into plastic tubes containing no additives; all blood samples were centrifuged twice at 2,500 g for 15 min at room temperature within 120 min of sampling. Plasma supernatants were transferred into cryotubes, frozen immediately and stored at −80°C until subsequent analyzes. Blood cultures and other microbiological tests were performed as part of and according to accepted clinical standards (30, 31). Blood culture and other microbiological tests procedures are described in more detail in Table S1 in Supplementary Material.

### Analysis of Plasma Mediator Levels of Soluble Mediators

Plasma levels of 38 soluble mediators were determined by Luminex^®^ analyzes (R&D Systems, Abingdon, UK) (32), including (i) six interleukins (ILs)—IL-1-β, IL-6, IL-8/CXCL8, IL-10, IL-12p70, and IL-1 receptor antagonist (IL-1ra); (ii) seven chemokines—CCL2/4/5/11 and CXCL5/10/11; (iii) six growth factors—granulocyte colony-stimulating factor (G-CSF), granulocyte macrophage colony-stimulating factor (GM-CSF), vascular endothelial growth factor, thrombopoietin (TPO), hepatocyte growth factor (HGF), and leptin; (iv) three immunomodulatory cytokines—interferon-gamma, CD40 ligand, and tumor necrosis factor alpha (TNF-α); (v) four soluble adhesion molecules P-selectin, E-selectin, intracellular adhesion molecule-1 (ICAM-1), and vascular cell adhesion molecule-1 (VCAM-1); and (vi) eight MMP-1/2/3/7/8/9/12/13 as well as four tissue inhibitors of metalloproteinases (TIMP) 1/2/3/4. In addition, the plasma level of IL-18 was determined by enzyme-linked immunosorbent assay (MBL Co. Ltd., Japan).

### Statistical and Bioinformatical Analyzes

Descriptive statistics were used to characterize the groups both generally as well as for initial comparison of soluble mediators in the groups. To assess the differences, we used the Mann–Whitney *U* test for continuous variables and the χ^2^ test for categorical variables (33). For a more comprehensive analysis, we selected those soluble mediators showing a significant difference and fitted a logistic regression model with the groups as outcomes and the mediators as predictors adjusted according to both age and organ failure (34). We estimated both a univariate model for each of the selected mediators and a fully adjusted model that included all selected predictors. Additionally, we performed unsupervised hierarchical clustering analyzes with complete linkage and Euclidean distance (27, 35). For both the logistic regression and cluster analyzes, the mediator values were log_10_- and *Z*-transformed (36). In this study, as well as in other reports of cluster analysis, we have found that the combination of Euclidean distance and complete linkage gives the best homology between mediator concentrations and the most compact clusters when running the clustering analysis (27–29).

Clustering analyzes were carried out using J-Express (MolMine AS, Bergen, Norway) (35). Other statistical analyzes were performed using Graph Pad Prism 5 (Graph Pad Software, Inc., San Diego, CA, USA) and SPSS (Version 20, release 20.0.0.2, IBM Corporation).

The general significance level was set at 0.05. To take into account multiple testing effects for the initial group comparison, we used the Bonferroni adjustment leading to the marginal level of 0.0014 (35 comparisons) (37, 38).

## Results

### Clinical Characteristics of 80 Patients With an Identified Cause of Bacterial Infections

A total of 80 patients had proven bacterial infection, with bacteremia confirmed in 42 patients (52.5%) by blood culture analyzes, and in 38 patients (47.5%) proven negative by blood culture analyzes, but confirmed bacterial infection by other tests. There were 37 (46.5%) and 38 (47.5%) patients with Gram-negative and Gram-positive bacterial infections, respectively, and 5 (6%) patients with mixed Gram-positive and Gram-negative bacterial infections. A complete list of all causes of bacterial infection in the study population is given in Table S1 in Supplementary Material.

The study patients were divided into two major subsets, based on whether bacteremia was detectable or not. Comparison of the clinical characteristics of these two subsets are shown in Table 1. The urinary tract and respiratory tract were the most common infection sites, whereas soft tissue, central nervous system, and abdominal infections as well as endocarditis were less common. A majority of patients with proven bacterial infection (52/80, 65%) presented with a total increase in the SOFA score of ≥2, and thus fulfilled the criteria for the new sepsis definition. Fifty-nine% (47/80) of patients required hospitalization in either a high-dependency unit or an ICU, the other 41% (33/80) were treated on a normal medical ward during their entire hospital stay. The in-hospital mortality rate was low for both groups with or without detectable bacteremia (a total of four patients corresponding to 5%). Antibiotic therapy was initiated within 2 h from admission for the vast majority of patients (74/80, 92.5%), within 3 h for 5/80 (6.3%), and one patient within 4 h. The initial antibiotic treatment was considered to be adequate and covered the identified infective pathogen for all, but four patients.

**Table 1:** Clinical characteristics for patient groups with and without bacteremia.

	With bacteremia (*n* = 42)	Without bacteremia (*n* = 38)	*p*-Value^d^
Age (years)^a^	72 (16–96)	60 (20–88)	**0.011**
Sex (female)^b^	21 (50%)	22 (58%)	0.479
Clinical findings at hospitalization^b^			
Temperature (°C)	38.8 (36.9–41.4)	38.6 (36.6–41.5)	0.780
Heart rate (/min)	108 (67–140)	112 (75–136)	0.900
Respiratory frequency (/min)	24 (15–60)	25 (11–53)	0.552
Systolic BP (mmHg)	120 (65–191)	126 (86–160)	0.338
Organ failure^b,c^			
Total SOFA ≥2	36 (85%)	16 (42%)	**<0.001**
Any failure SOFA ≥2	28 (67%)	10 (26%)	**0.010**
Respiratory failure	17 (40%)	10 (26%)	0.181
Hypotension	9 (21%)	3 (8%)	0.090
Bleeding disorder	5 (12%)	1 (3%)	0.107
Renal failure	7 (17%)	3 (8%)	0.236
Liver failure	2 (5%)	1 (3%)	0.616
CNS failure	3 (7%)	2 (5%)	0.729
Infection site^b^			**0.003**
Urinary tract	20 (48%)	17 (45%)	
Respiratory	5 (12%)	13 (34%)	
Abdominal	3 (7%)	0	
Soft tissue	4 (10%)	8 (21%)	
CNS	4 (10%)	0	
Endocarditis	6 (14%)	0	
Bacteria^b^			**0.044**
Gram-negative	25 (60%)	12 (32%)	
Gram-positive	15 (36%)	23 (61%)	
Mixed Gram-pos./neg.	2 (5%)	3 (8%)	
Microbe not sensitive to initial treatment	3 (7%)	1 (3%)	0.342
Hospitalization at^b^			0.103
HDU	23 (55%)	16 (42%)	
Medical ward	13 (31%)	20 (53%)	
ICU	6 (14%)	2 (5%)	
Hospitalization (days)^b^	7.5 (2–55)	7 (2–50)	0.399
Mortality^b^	1 (2%)	3 (7%)	0.259
Routine biochemistry^a^			
Hgb (g/dL)	12.5 (8.7–15.4)	13.0 (9.2–17.5)	0.429
WBC (10^9^/L)	13.6 (2.0–27.9)	14.2 (4.9–46.6)	0.776
Neutrophils (10^9^/L)	12.1 (1.6–24.3)	12.1 (4.1–41.1)	0.866
CRP (mg/L)	175 (3–457)	158 (7–538)	0.071
Creatinine (μmol/L)	108 (48–706)	70 (27–475)	**0.001**

*^a^Median (min–max), Mann–Whitney *U* test*.

*^b^*N*(%), χ^2^ test*.

*^c^Defined as increase in sequential (sepsis-related) organ failure assessment score ≥2, sepsis induced (pre-existing, stable organ failure not included)*.

*^d^Significant values are displayed in bold*.

### Plasma Levels of Soluble Mediators Differ in Patients With, Compared With Those Without, Bacteremia

**Figure 2:**
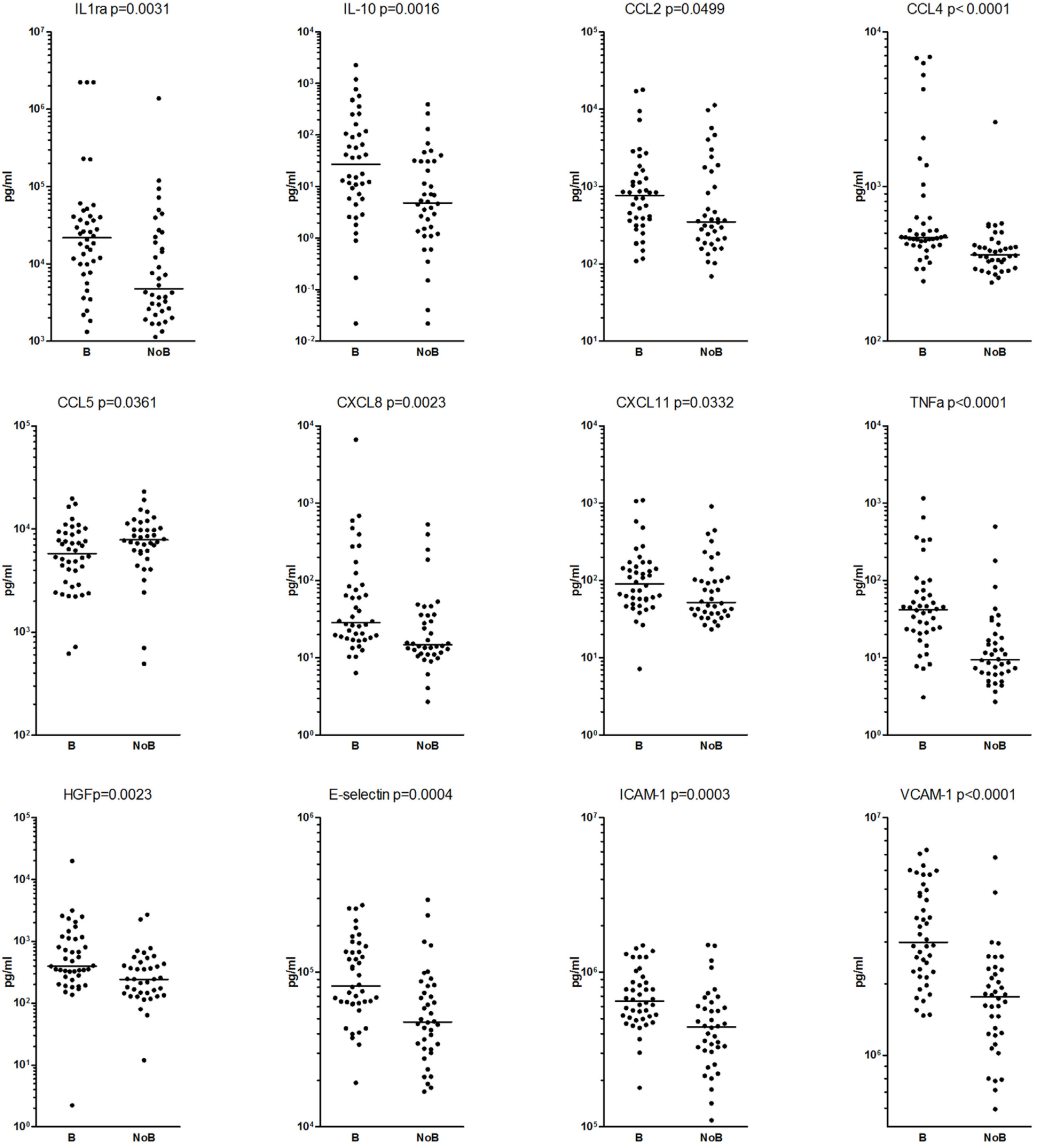

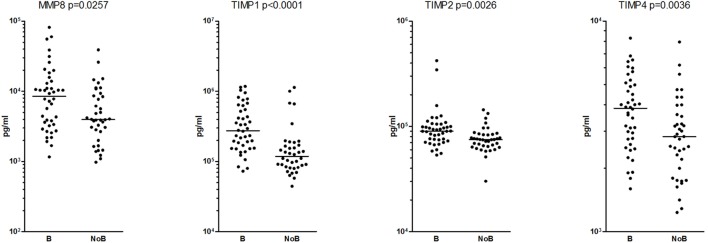
The mediators with significant differences between the patients with (B) and without (NoB) bacteremia are presented and each dot represent one patient. The Mann–Whitney *U* test was used for the comparisons, *p* < 0.05 were regarded as significant and *p* < 0.0014 were also significant after Bonferroni correction.

We compared the plasma levels of 39 soluble mediators in patients with and without bacteremia. Four soluble mediators (IL-12p, MMP-7/12, and TIMP-3) were undetectable for either all or most patients and therefore were not included in statistical analyzes (Table 2). For 16 of the remaining 35 soluble mediators, we found statistically significant differences in plasma levels (*p* < 0.05) between the two patient groups. After Bonferroni correction, the plasma levels of only 6 of these 16 soluble mediators remained significantly different (*p* < 0.0014) between the two patient groups (Table 2; Figure 2). These six soluble mediators were heterogeneous in biological function and included; the pro-inflammatory mediators CCL4 and TNF-α; the three soluble adhesion molecules VCAM-1, ICAM-1, and E-selectin that also have anti-inflammatory properties; and the protease inhibitor TIMP-1.

**Table 2:** Mediators in patients with and without bacteremia; the median value and range are given.

Mediator	Patients		Mann–Whitney	Logistic regression			
			
				Univariate		Multivariate^a^	
					
	Without bacteremia	With bacteremia	*p*-Value	OR 95%CI	*p*-Value	OR 95% CI	*p*-Value
**Interleukins**							
IL-1ra^b^	4.7 (1.1, 1,376.0)	21.7 (1.3, a.d.)	0.0031				
IL-1β^c^	1.4 (0.1, 61.0)	3.1 (n.d., 64.3)	0.0763				
IL-6^b^	0.11 (0.08, 119)	0.15 (0.03, 13.8)	0.3187				
IL-10^c^	4.8 (n.d., 394.7)	26.9 (n.d., 2,256)	0.0016				
IL-18^b^	0.29 (0.10, 2.3)	0.32 (0.08, 20.0)	0.5280				

**Chemokines**							
CCL2^b^	0.35 (0.07, 11.3)	0.77 (0.11, 17.8)	0.0499				
CCL4^b^	0.36 (0.24, 2.6)	0.47 (0.24, 6.9)	**<0.0001**	**4.3 (1.4, 13.2)**	**0.0100**	1.8 (0.6, 5.2)	0.288
CCL5^b^	7.8 (0.49, 23.0)	5.8 (0.62, 19.7)	0.0361				
CCL11^b^	0.20 (0.05, 0.57)	0.18 (0.07, 17.9)	0.6610				
CXCL5^b^	0.18 (0.09, 0.77)	0.26 (0.03, 5.26)	0.0881				
CXCL8^c^	14.7 (2.7, 529.8)	28.4 (6.34, 6,634)	0.0023				
CXCL10^c^	158.0 (10.77, 5,341)	296.7 (41.68, 4,942)	0.0511				
CXCL11^c^	51.9 (23.23, 907.7)	90.5 (7.14, 1,091)	0.0332				

**Immunomodulatory cytokines**							
TNF-α^c^	9.4 (2.68, 497.1)	42.7 (3.07, 1,157)	**<0.0001**	**3.9 (1.9, 7.9)**	**<0.0001**	1.2 (0.4, 3.6)	0.761
IFN-γ^c^	3.7 (n.d., 22.91)	2.8 (n.d., 14.2)	0.2381				
CD40L^b^	0.88 (0.3, 2.7)	0.83 (0.4, 2.0)	0.4408				

**Growth factors**							
VEGF^b^	0.26 (0.07, 0.4)	0.21 (0.08, 0.5)	0.1130				
TPO^b^	0.84 (0.4, 7.0)	1.00 (0.5, 3.1)	0.1255				
HGF^b^	0.24 (0.01, 2.7)	0.4 (n.d., 20.0)	0.0023				
G-CSF^b^	0.18 (0.04, 31.6)	0.63 (0.07, 34.0)	0.1417				
GM-CSF^c^	0.09 (n.d., 5.98)	0.33 (n.d., 9.36)	0.3332				
Leptin^b^	10.5 (1.0, 172.4)	13.8 (0.6, 11.5)	0.8059				

**Adhesion molecules**							
E-Selectin^b^	47.6 (16.9, 293.7)	81.6 (19.2, 270.8)	**0.0004**	**2.5 (1.4, 4.3)**	**0.0010**	1.2 (0.6, 2.5)	0.5120
P-Selectin^b^	23.0 (10.4, 39.8)	23.2 (11.12, 39.0)	0.4495				
ICAM-1^d^	0.44 (0.11, 1.50)	0.65 (0.17, 1.49)	**0.0003**	**3.0 (1.6, 5.5)**	**<0.0001**	1.5 (0.7, 3.1)	0.2100
VCAM-1^d^	1.8 (0.6, 6.8)	3.0 (1.5, 7.3)	**<0.0001**	**5.3 (2.4, 11.8)**	**<0.0001**	**3.4 (1.3, 8.8)**	**<0.0001**

**Matrix metalloproteases and inhibitors**							
MMP-1^b^	0.56 (0.14, 7.6)	0.58 (0.17, 5.0)	0.4380				
MMP-2^b^	0.05 (0.03, 53.1)	45.8 (28.1, 52.0)	0.6062				
MMP-3^b^	9.4 (2.5, a.d.)	12.6 (3.9, a.d.)	0.0550				
MMP-8^b^	3.9 (0.97, 38.7)	8,515 (1.2, 81.4)	0.0257				
MMP-9^b^	3.9 (n.d., 122.9)	2.0 (n.d., 73.1)	0.1328				
MMP-13^b^	0.46 (0.04, 0.9)	0.53 (0.02, 4.2)	0.6161				
TIMP-1^d^	0.12 (0.04, 1.13)	0.27 (0.07, 1.17)	**<0.0001**	**2.6 (1.4, 4.5)**	**0.0030**	1.0 (0.4, 2.6)	0.5950
TIMP-2^b^	74.9 (30.1, 143.6)	89.9 (53.3, 419.3)	0.0026				
TIMP-4^b^	2.8 (1.2, 8.0)	3.8 (1.6, 8.3)	0.0036				

*^a^Model including all the six mediators; age and organ failure are not included as predictors, as they did not have major impact on the mediators OR and p-values. ^b^ng/ml, ^c^pg/ml, ^d^μg/ml*.

*n.d.: not detected; a.d.: above detection range*.

*Mann–Whitney U test is used for comparison, p < 0.05 is regarded as significant, p < 0.0014 also significant after Bonferroni correction are marked in bold*.

Univariate logistic regression showed significant results for all of these six soluble mediators (Table 2). In a multivariate logistic regression model that included all six soluble mediators, VCAM-1 still showed statistically significant differences in plasma levels between patients with, compared with those without bacteremia, whereas age and organ failure did not impact the logistic regression model, and were not included in the final model.

The 10 soluble mediators showing statistically significant differences in plasma levels only before Bonferroni correction were also heterogeneous in biological function and included; the anti-inflammatory mediators IL-1ra, IL-10, and HGF; the pro-inflammatory chemokines CCL2, CCL5, CXCL8, and CXCL11, MMP-8; and the protease inhibitors TIMP-2 and TIMP-4 (Table 2; Figure 2).

### The Systemic Soluble Mediator Profile Shows Only Minor Differences Between Patients With Gram-Positive and Those With Gram-Negative Bacterial Infections

We compared the soluble mediator levels between patients with documented Gram-positive bacterial infections and those with Gram-negative bacterial infections, which also included patients with and without bacteremia. Results showed only minor differences in plasma profiles between the Gram-positive and Gram-negative bacterial infection groups. We observed significant differences only for CCL4 (*p* = 0.0057), CXCL5 (*p* = 0.0452), CXCL10 (*p* = 0.0393), and leptin (*p* = 0.0150), with higher levels of CCL4 and CXCL5 in the Gram-negative group, and with higher levels of CXCL10 and leptin in the Gram-positive group. However, these differences were not significant after Bonferroni correction.

### Identification of a Patient Subset With a Low Frequency of Bacteremia by Unsupervised Hierarchical Clustering of All Mediators

**Figure 3:**
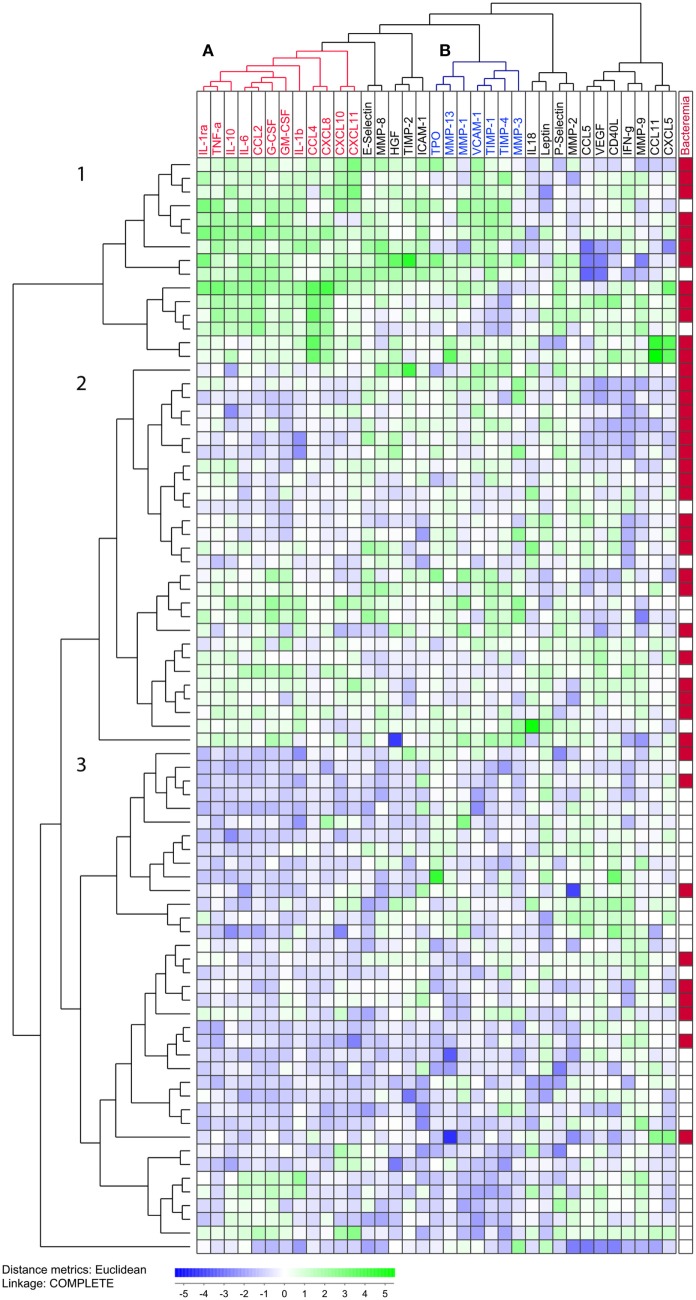
Unsupervised hierarchical clustering analysis of plasma levels for 35 detectable mediators, and an analysis including all 80 patients. The concentrations were log_10_ converted and *Z*-transformed standardized before unsupervised hierarchical clustering. Euclidian correlation test with complete linkage were used for the clustering analysis. The heat-map displays a small square for each mediator for each patient, and each squares colors displays its concentration compared with mean and corrected for SD. The mediators are clustered horizontally and a tree is formed at the top of the figure that display the covariation of different mediators, and to the left a tree is formed where patients with similar mediator covariation cluster together. To the right, we have added clinical information. We see that most mediators cluster close to (i.e., have a similar variation as) biologically related mediators and most of the mediators that differ between the patients are grouped in or between cluster **A** (red) and **B** (blue). The patients are clustered vertically and form three main clusters, 1, 2, and 3. The patients with bacteremia are marked in red in the right column. Cluster 3 with the lowest mediator levels included a smaller proportion of patients with bacteremia.

We performed unsupervised hierarchical clustering including all 35 soluble mediators and all 80 patients with sepsis with an identified bacterial cause. This was performed to determine the covariation of both patients and soluble mediators (Figure 3).

Results showed three main clusters of soluble mediators with similar biological functions: (i) cluster A which consisted of cytokines that play an important role in the regulation of the initial inflammatory response (IL-1b, TNF-α, IL-1ra, IL-10, and IL-6), growth factors (G-CSF and GM-CSF) and CXCL chemokines (CXCL8/10/11); (ii) cluster B composed of VCAM-1, TPO and most of the proteases and their inhibitors; and (iii) a third cluster which included adhesion molecules (E-selectin and ICAM-1), as well as proteases and protease inhibitors (MMP-8 and TIMP-2).

Furthermore, findings revealed three main patient clusters: upper cluster 1 which showed generally high-cytokine levels; middle cluster 2 with varied and intermediate, and lower cluster 3 which showed generally low-cytokine levels. Patient clusters 1 and 2 had the highest soluble mediator levels, whereas cluster 3 had lower soluble mediator levels, as well as a significantly lower frequency of patients with bacteremia (χ^2^ test, *p* < 0.0001).

### Identification of a Patient Subset With a Low Frequency of Bacteremia and Low-Serum Soluble Mediator Levels by Hierarchical Clustering, Based on the Six Mediators Showing the Highest Significant Differences in Plasma Levels Between Patients With and Those Without Bacteremia

**Figure 4:**
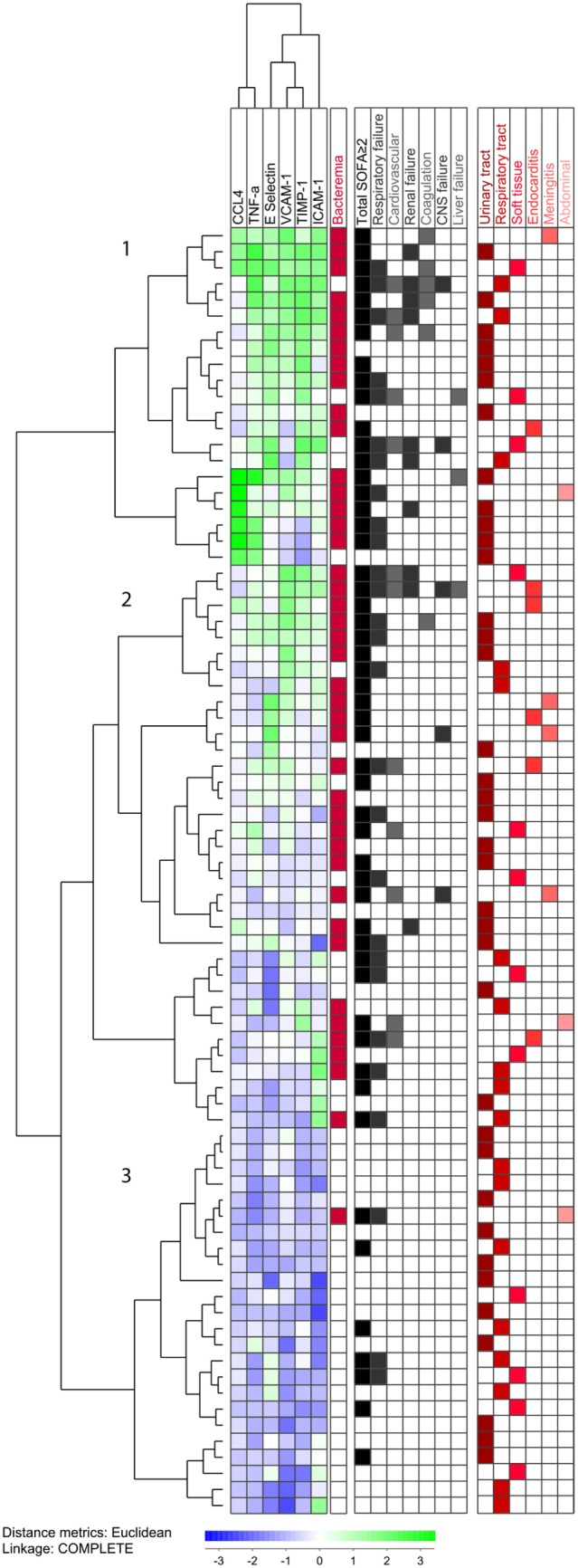
Unsupervised hierarchical clustering analysis of plasma levels for the six most significant mediators. All 80 patients were included. The concentrations were log_10_ converted and *Z*-transformed standardized before unsupervised hierarchical clustering. Euclidian correlation test with complete linkage were used for the clustering analysis. The heat-map displays a small square for each mediator for each patient, and each squares colors displays its concentration compared with mean and corrected for SD. The mediators are clustered horizontally and a tree is formed at the top of the figure that display the covariation of different mediators, and to the left a tree is formed where patients with similar mediator covariation cluster together. To the right, we have added clinical information. The mediators are clustered horizontally, while the patients are clustered vertically and form the three main clusters, 1, 2, and 3. We find that both bacteremia and organ failure are found in the clusters containing the patients with higher and intermediate levels of inflammatory mediators. The patients with bacteremia are marked in red in the right column. We see that the lower subcluster (3) contains a lower proportion of the bacteremia patients, and 41/42 (98%) of the bacteremia patients are clustered in the two higher (1 and 2) subclusters. The right part of the figure shows organ failure(s) and infectious site for each individual patient. The patients with total sequential (sepsis-related) organ failure assessment (SOFA)-score ≥2 identify the patients fulfilling the new sepsis criteria (65% of the patients). We see a higher proportion of organ failures in the two upper patient clusters, while no clear pattern identifies the infection site.

In order to better differentiate between patients with and those without bacteremia, clustering analysis was conducted based solely on those mediators showing statistically significant differences in plasma levels between the two groups after Bonferroni correction (Figure 4). The analysis included the pro-inflammatory cytokines TNF-α and CCL4, as well as the immunoregulatory protease inhibitor TIMP-1 and the soluble adhesion molecules VCAM-1, ICAM-1, and E-selectin. This analysis identified a patient subset with generally low-plasma levels and a very low frequency of patient subsets with bacteremia (Figure 4; patient subcluster 3; 1/24 patients having bacteremia), and two patient subsets showing high levels of several soluble mediators and a high frequency of patients with bacteremia (patient subclusters 1 and 2; 41/66 patients having bacteremia). Thus, patient subclusters 1 and 2 included 41 of the 42 patients with bacteremia (98%), whereas the lower subcluster 3 included only one (2%) of the bacteremia patients, as well as the majority of patients without bacteremia (23/38, 61%). This difference is highly significant (χ^2^ test, *p* < 0.0001).

Finally, the three subclusters showed no difference with regard to the site of infection, but the frequency of patients with organ failure (total SOFA score ≥2) was lowest for the “low- bacteremia” subcluster 3 (Figure 4; 7/24 versus 45/66, χ^2^ test, *p* = 0.0009). Cluster 1 (patients with the highest soluble mediator levels) included a majority of the patients with bleeding disorders, whereas cluster 3 (patients with the lowest soluble mediator levels) included predominantly respiratory failure as organ failure with a SOFA score of ≥2.

## Discussion

Previous studies investigated cytokine profiles in patients with sepsis (8, 9, 22–24), but in the present study we examined a broader profile by including not only a larger number of cytokines, but also adhesion molecules and MMPs. In our study, we found six of the soluble mediators analyzed that differentiated between patients with and without bacteremia: TNF-α, CCL4, TIMP-1, VCAM-1, ICAM-1, and E-selectin. Ten other soluble mediators also showed differences, but with lower significance. VCAM-1 was the only mediator that consistently showed significant differences in univariate and multivariate logistic regressions, and with the strongest association with bacteremia. The unsupervised hierarchical clustering model including all soluble mediators showed that soluble mediators with similar biological properties generally clustered close together. By combining the six most significant pro-inflammatory soluble mediators in the unsupervised hierarchical clustering model, we were able to differentiate 98% of patients with bacteremia, with the majority of patients with organ failure also included in this group.

Our prospective cohort showed similar characteristics (infection sites, microbes, and disease severity) as the sepsis patients included in a previous observational study at our hospital, thus confirming our patients are representative of sepsis patients in our region (39). Furthermore, the low percentage of patients needing ICU admission in our study suggests that our cohort is representative of typical sepsis cases presenting to emergency departments, and not only of sepsis in an ICU setting. In order to investigate in more detail the inflammatory response to various bacterial infections, we studied those patients with proven bacterial infections only, and no culture-negative patients were included (12).

Bacteremia has previously been associated with disease severity and organ failure (11, 40–42). Therefore, it is not surprising that we obtained similar results in our patient cohort. Disease severity is graded according to organ-failure using the SOFA score (4). The rates of bacteremia was shown in an early prospective study to vary according to the degree of severity of sepsis where bacteremia was found in 17% of patients with sepsis, 25% of patients with severe sepsis, and 69% of patients with septic shock (43). A key question that arises from our study is whether the increased inflammatory response seen was directly related to bacteremia, or was only associated with bacteremia through the degree of disease severity. Our results from the multiple regression analysis implied that bacteremia was an independent factor, as the six examined soluble mediators analyzed remained significant after correcting for patients with an increased total SOFA score of ≥2. Our hierarchical clustering model (Figure 4) showed that both bacteremia and organ failure were found in patients with higher levels of inflammatory mediators (subclusters 1 and 2). Only one patient with bacteremia was found among patients with the lowest levels of inflammatory mediators (subcluster 3), while in contrast there still were several patients with organ failure in this cluster.

Mortality rates obtained in our study were low, similar to previous comparable studies (44, 45), which makes it difficult to draw conclusions regarding mortality in our cohort. As appropriate antibiotic treatment is crucial for survival in sepsis (46), more detailed biological characterization with identification of patients likely to have bacteremia could help to guide antibiotic treatment.

Systemic levels of single mediators in sepsis patients have been examined extensively in previous studies (5). As our study was limited to a cohort size of only 80 patients and included 35 possible inflammatory mediators, one should be careful using univariate and multivariate logistic regression analyzes of our results. Despite this, VCAM-1 stands out as a possible important marker for bacteremia in contrast to results from an earlier study (47). TNF-α is one of the most important pro-inflammatory cytokines during an acute septic reaction (5, 6, 13), as well as inducing the expression of adhesion molecules that help activate and recruit leukocytes during an inflammatory response (15, 48). The chemokine CCL4 is also an active player in the inflammatory response and chemotaxis during sepsis (5, 49, 50). In addition, MMPs as well as the balance between MMPs and their inhibitors, have been found to influence progression of inflammatory disorders (16).

Cytokine profiling in sepsis patients seems to impact the prognostic evaluation of these patients, as shown in previous studies conducted in an ICU setting (8, 26). Our study suggests that not only cytokines, but also soluble adhesion molecules, proteases, and their inhibitors should be included in such profiling. This strategy for prognostic evaluation should also be investigated in other cohorts of sepsis patients, e.g., patients in an ICU setting, to determine the possible use of prognostic evaluation with regard to mortality risk (39). Using the new sepsis definition, 35% of patients in this study did not fulfill the criterion of an increased total SOFA score of ≥2. However, in an emergency department setting cytokine profiling might help stratify patients hospitalized with serious infections (4).

We expected to find a lager difference in inflammatory response between Gram-positive and Gram-negative bacterial infections, but in contrast to previous findings (51, 52), we found no major differences among the inflammatory mediators. This could be explained by the multiple infection sites present in our cohort, as opposed to studies examining patients with, for example, abdominal infections only (53). All the mediators that showed minor differences CCL4, CXCL5, CXCL10 and leptin all exert pro-inflammatory effects (6, 54, 55).

Use of unsupervised hierarchical clustering is a novel methodology that previously was used mainly in studies of gene expression profiles. However, this methodological strategy may also become useful for cytokine profiling (27–29, 35). Our results showed a discriminative pattern when analyzing the six most important pro-inflammatory mediators included in this study. The two upper clusters compromised patients with the highest levels of the inflammatory mediators, including the vast majority of the patients with bacteremia which probably reflects a biologically stronger inflammatory response in patients with bacteremia. Clustering analysis of soluble mediators could contribute to a better understanding of the inflammatory response in sepsis and its prognostic impact (2, 4).

Our study suggests that broader profiling of soluble mediators should be further investigated as possible biomarkers in patients with sepsis (4). This strategy could prove useful to distinguish between patients with and those without bacteremia during the initial phase of sepsis. However, confirmation of the applicability of this strategy in future clinical studies and the development of new bioinformatical tools are needed before this approach can be introduced in routine clinical practice.

## Ethics Statement

The study was approved by the regional Ethics Committee (REK Vest Norway, number 103-2013), and have been performed in accordance with the ethical standards laid down in the 1964 Declaration of Helsinki and its later amendments. Patients were included after written consent.

## Author Contributions

KM main author. Design and planning, including study participants and manuscript writing. SS, DM, HRF, and HF including study participants and manuscript editing. JA statistical evaluation and manuscript editing. ØB and HR idea for the study, design, and planning, manuscript editing.

## Conflict of Interest Statement

The authors declare that the research was conducted in the absence of any commercial or financial relationships that could be construed as a potential conflict of interest.
