# Supplementary material for: Inflammatory Mediator Profiles Differ in Sepsis Patients With and Without Bacteremia
Source: Front Immunol. 2018 Apr 6;9:691. doi: 10.3389/fimmu.2018.00691 (PMC5897503; doi:10.3389/fimmu.2018.00691)
Supplement: Supplementary file 1 [file data_sheet_1.DOCX]

**Supplementary table 1.** The table presents the microbial findings for individual patients, each line representing one patient. Procedures for blood culture are given below^1^.

| **Microbe, blood culture** | **Microbe, other medium** |
| --- | --- |
| Escherichia coli | Escherichia coli (Urine culture) |
| Escherichia coli | Escherichia coli (Urine culture) |
| Escherichia coli | Escherichia coli (Urine culture) |
| Escherichia coli | Escherichia coli (Urine culture) |
| Klebsiella pneumoniae | Klebsiella pneumoniae (urine culture) Escherichia coli (urine culture) |
| Klebsiella pneumoniae/Proteus mirabilis |  |
| Enterobacter cloacae |  |
| Bacteroides fragilis |  |
| Escherichia coli |  |
| Fusobacterium necrophorum |  |
| Escherichia coli (ESBL) |  |
| Escherichia coli (ESBL) |  |
| Escherichia coli | Escherichia coli (urine culture) |
| Kingella kingae |  |
| Escherichia coli |  |
| Neisseria meningitidis |  |
| Klebsiella pneumoniae |  |
| Bacteroides ovatus and uniformis |  |
| Escherichia coli | Escherichia coli (urine culture) |
| Escherichia coli | Escherichia coli (urine culture) |
| Escherichia coli |  |
| Escherichia coli | Escherichia coli (urine culture) |
| Escherichia coli |  |
| Escherichia coli | Escherichia coli (urine culture) |
| Escherichia coli |  |
| Streptococcus constellatus/ Actinobaculum schaalii |  |
| Staphylococcus aureus /Klebsiella oxytoca |  |
| Staphylococcus aureus | Staphylococcus aureus (Tissue biopsy) |
| Streptococcus pneumoniae | Streptococcus pneumoniae (Positive Urinary Antigen Test) |
| Staphylococcus aureus |  |
| Streptococcus pyogenes (group A-streptococci) |  |
| Streptococcus dysgalactiae subspecies equisimilis, Staphylococcus aureus |  |
| Staphylococcus aureus |  |
| Staphylococcus aureus |  |
| Staphylococcus aureus |  |
| Streptococcus pneumoniae |  |
| Streptococcus pneumoniae |  |
| Streptococcus pneumoniae | Escherichia coli (urine culture) |
| Staphylococcus aureus | Staphylococcus aureus (endotracheal aspirate culture) |
| Streptococcus pneumoniae | Streptococcus pneumoniae (cerebrospinal fluid culture) |
| Streptococcus pneumoniae | Streptococcus pneumoniae (cerebrospinal fluid culture) |
| Streptococcus intermedius; Pavimonas micra; Fusobacterium spp. | Multiple microbes (pus) |
| **Microbe, blood culture** | **Microbe, other medium** |
|  | Escherichia coli (urine culture) |
|  | Escherichia coli (urine culture) |
|  | Streptococcus pyogenes / Staphylococcus aureus (Pus) |
|  | Staphylococcus saprophyticus (Urine culture) |
|  | Streptococcus pneumoniae **(**Positive Urinary Antigen Test**)** |
|  | Streptococcus pneumoniae **(**Positive Urinary Antigen Test) |
|  | Escherichia coli (urine culture) |
|  | Streptococcus pneumoniae **(**Positive Urinary Antigen Test) |
|  | Streptococcus pyogenes (soft tissue) |
|  | Escherichia coli (urine culture) |
|  | Actinobaculum schaalii (urine culture) |
|  | Escherichia coli (urine culture) |
|  | Streptococcus pneumoniae /Strep. milleri group, Staphylococcus aureus (endotracheal aspirate culture) |
|  | Streptococcus pneumoniae **(**Positive Urinary Antigen Test) |
|  | Staphylococcus aureus (wound /tissue) |
|  | Clostridium difficile (Clostridium toxin A) |
|  | Staphylococcus epidermidis (wound and periprosthetic tissue culture) |
|  | Streptococcus pyogenes (pus) |
|  | Escherichia coli (Urine culture) |
|  | Beta-hemolytic Streptococcus (Urine culture) |
|  | Streptococcus pyogenes (Soft tissue sample) |
|  | Escherichia coli (Urine culture) |
|  | Escherichia coli (Urine culture) |
|  | Staphylococcus aureus; Citrobacter koseri, Finegoldia magna, Anaerococcus (Soft tissue sample) |
|  | Escherichia coli (Urine culture) |
|  | Streptococcus pneumoniae **(**Positive Urinary Antigen Test) |
|  | Streptococcus pneumoniae **(**Positive Urinary Antigen Test) |
|  | Streptococcus pneumoniae **(**Positive Urinary Antigen Test) |
|  | Serratia marcescens and Enterococcus faecalis (Urine culture) |
|  | Streptococcus pneumoniae **(**Positive Urinary Antigen Test) |
|  | Escherichia coli and Enterococcus spp. (Urine culture) |
|  | Escherichia coli (Urine culture) |
|  | Streptococcus intermedius (Pleural fluid) |
|  | Streptococcus dysgalactiae (Throat swab) |
|  | Escherichia coli (Urine culture) |
|  | Streptococcus pyogenes (Tissue sample) |
|  | Escherichia coli, ESBL (Urine culture) |
|  | Proteus mirabilis (Urine culture) |

^1^Automated blood culture systems BacT/ALERT (bioMérieux, Marcy l'Etoile, France) was used for microbial growth detection. Blood culture broths (BacT/ALERT® FA and BacT/ALERT® FN; bioMérieux Inc., Durham, NC, USA) identified as positive by the BacT/ALERT automated blood culture instrument were analysed using MALDI-TOF MS (Daltonics GmbH, Bremen, Germany) for direct species identification.
